# Fatal avian influenza A(H5N1) infection in a 36‐week pregnant woman survived by her newborn in Sóc Trăng Province, Vietnam, 2012

**DOI:** 10.1111/irv.12614

**Published:** 2019-02-27

**Authors:** Tuan Van Le, Lan T. Phan, Khanh H. K. Ly, Long T. Nguyen, Hieu T. Nguyen, Ngan T. T. Ho, Tung X. Trinh, Nguyen N. Tran Minh

**Affiliations:** ^1^ World Health Organization Vietnam Country Office Ho Chi Minh Vietnam; ^2^ Ho Chi Minh City Pasteur Institute Ho Chi Minh Vietnam; ^3^ General Department of Preventive Medicine Ha Noi Vietnam

**Keywords:** avian influenza, H5N1, mother‐to‐child, transmission

## Abstract

**Background:**

Reports of pregnant women infected with avian influenza are rare. Studies showed that A/H5N1 virus can penetrate the placental barrier and infect the fetus. Of six documented cases, four died and two survivors had a spontaneous abortion.

**Objectives:**

We report a clinical, outcome and epidemiological characteristics of a 36‐week pregnant woman infected with A/H5N1 and her newborn in Soc Trang province of Vietnam in 2012.

**Methods:**

Epidemiological and laboratory investigations were conducted. Clinical manifestations, progress, treatment and outcome of the case‐patient and her newborn were collected. Human tracheal aspirate, throat swab and serum specimens were tested for influenza A/H5N1, A/H3N1, A/H1N1pdm09 and B by real‐time RT‐PCR and genome sequencing. Poultry throat and rectal swabs were tested by PCR and virus isolation.

**Results:**

Case‐patient hospitalized with high fever and cough, and died after onset 6 days. She continuously slaughtered sick poultry 5 days before illness onset. Clinical manifestation showed rapid progressive severe pneumonia. Her tracheal aspirate sample was positive influenza A/H5N1 virus. Her new‐born was delivered by caesarean section with low birth weight and early onset pneumonia, however fully recovered after 16 days treatment. Neonate's throat swabs and paired serum samples tested negative for influenza A/H5N1. Clade 1.1 A/H5N1 virus was detected in poultry samples, was same clade and highly homogenous with the virus was detected in the mother.

**Conclusions:**

This was the first documented a live birth from a pregnant woman infected with influenza A/H5N1 virus. Intensive studies are needed to better understand mother‐to‐child transmission of influenza A/H5N1 virus.

## 1 BACKGROUND

The outbreak of avian influenza A(H5N1) virus in poultry and human infection emerged in Indonesia, Thailand and Vietnam during 2003‐2005. Its severe impact has been reported by the United Nations Food and Agriculture Organization and the World Bank, which estimated that Vietnam lost up to 1.8% of their gross domestic product as a result.^1^ Since then Vietnam has reported over 3000 A(H5N1) poultry outbreaks,^2^ cases of human A(H5N1) infection and 64 deaths. Vietnam ranks second in the world in fatality and mortality of human avian influenza.^3^ From 2003 to 2012, Mekong and the Southeast regions reported human cases of A(H5N1) infection and deaths accounting for 27% and 45% of the entire country respectively. The case fatality rate (CFR) of the Southern region was nearly 1.7 times higher than the CFR in Vietnam.^4^

In January 27, 2012, the Sóc Trăng Provincial Preventive Medicine Center was informed about a pregnant patient with suspected avian influenza infection who was admitted to a provincial hospital with high fever and cough. On that day, the Ho Chi Minh City Pasteur Institute (HCMC PI) was also informed about this case. A day after, the provincial hospital reported that the pregnant patient had died; however, her baby was delivered by cesarean section alive but critically ill.

It was noted that pregnancy associated with A(H5N1) infection is very rare and little is known about A(H5N1) infection during pregnancy. Of the six cases that have been documented, four pregnant women died and two pregnant women who survived had spontaneous abortions.^5^ In addition, a recent study showed that the CFR for A(H5N1) infection in pregnant women was 10%‐25%, which is much higher than the general population with A(H5N1) infection at 0.3%‐1%.^6^

This case report describes the epidemiological characteristics, clinical progress and outcome of the Vietnamese pregnant woman (case‐patient) and her newborn. Results of the in‐depth outbreak investigation to trace the cause of the A(H5N1) infection have been presented.

## 2 METHODS

We conducted a case study of the clinical characteristics and the outcomes of the case‐patient who was pregnant at the time of diagnosis with avian influenza A(H5N1) infection and her newborn baby. An initial risk assessment was conducted and an in‐depth outbreak investigation carried out by a team of epidemiologists, clinicians, virologists and local veterinarians.

The World Health Organization (WHO) guidelines for investigation of human cases of avian influenza A(H5N1)^7^ were used to develop investigation tools. The medical records of the case‐patient were reviewed and the physicians who cared for the case‐patient were also interviewed.

The team collected the epidemiological data of the case‐patient and her newborn including the exposure history. Contact tracing was implemented where a list of contacts was developed and for close contacts that were identified were monitored. Contacts were defined as those who had contact with the case‐patient eg meeting with the case‐patient or had been exposed to the same source eg contact with poultry that was sick or had died such as raising or touching the poultry. Close contact was defined as anyone who directly came into contact with the case‐patient within 1 m eg the health care worker, providing bedside care, speaking with the case‐patient, touching the case‐patient and providing transportation for the case‐patient, as well as any household members who had slaughtered poultry that was either sick or touched poultry that had died, who had shared the same sleeping or eating space 1 day prior to the onset of the case‐patient's illness through to 14 days after the case‐patient's onset of illness.

The team conducted a field visit which included in‐depth interviews with the case‐patient's family and community members to collect information regarding illness and outbreaks among birds, poultry and animals. Avian influenza A(H5N1) virus infection case reports by provincial and district health centers and animal and poultry outbreak reports from local animal health stations were also collected and reviewed.

Clinical data were examined and these included clinical symptoms, treatment and outcome, and laboratory results of both the case‐patient and her newborn.

Human tracheal aspirate (from the case‐patient), throat swabs and serum specimens (from the newborn) were collected and sent to the HCMC PI, the national influenza center laboratory for testing: influenza A(H5N1); A(H3N1); A(H1N1) pdm09 viruses; and influenza B virus by real time reverse transcription‐polymerase chain reaction (RT‐PCR), and viral isolation and genome sequencing. The samples of the case‐patient and her newborn were also sent to the United States Centers for Disease Control and Prevention (CDC), Influenza Division to verify the test results. Throat swabs of close contacts were taken and tested for the avian influenza A(H5N1) virus to detect for further avian influenza human cases.

Poultry throat and rectal swabs were collected and tested for the avian influenza A(H5N1) virus by RT‐PCR and viral isolation at the laboratory of the Regional Animal Health Office and the National Center for Veterinary Diagnosis in Vietnam.

## 3 RESULTS

### 3.1 Clinical course and laboratory data: Case‐patient

The case‐patient was a 26‐year‐old rice farmer, without any history of chronic diseases who was pregnant at 36 weeks gestation. She had two antenatal examinations at the second and third trimester with no pregnancy complications noted. The seasonal influenza vaccine was not available in the case‐patient's hometown and as a result had not been vaccinated. The case‐patient had not visited any poultry outbreak site or live bird market in the 7 days prior to the onset of her symptoms.

**Figure 1:**
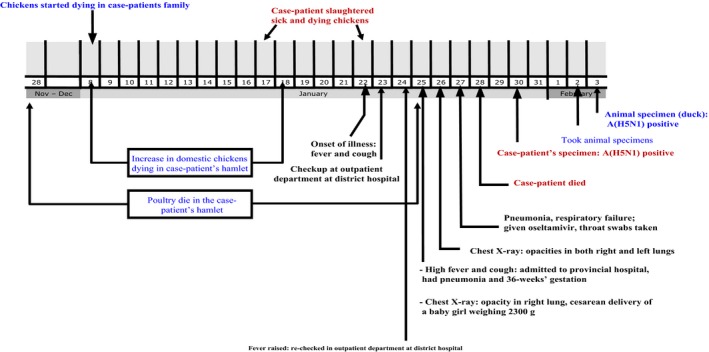
Time line on the human and poultry outbreak of A(H5N1) virus between November 2011 and February 2012 in Sóc Trăng Province, Vietnam

The case‐patient's hospital course and related information was collected from her medical records. She developed fever and cough on January 22, 2012 and had been treated by a local clinician on the same day. Two days later, she was treated at an outpatient department of a district hospital and then hospitalized at the provincial hospital on January 25, 2012 presenting with cough and high fever. The admission diagnoses were: pregnancy at 36 weeks gestation; oligohydramnios (amniotic fluid index = 5) and pneumonia. She was treated initially with antibiotics and antipyretics. The doctors then decided to deliver her baby by cesarean section (Figure 1).

**Figure 2:**
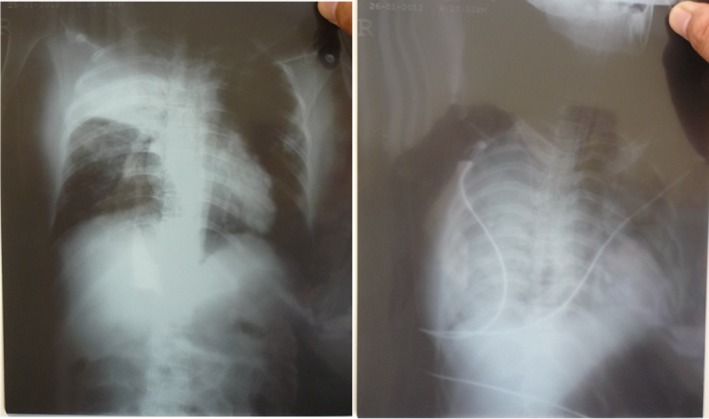
Progression of pulmonary disease in the chest X‐ray of the case‐patient (left: day 4 of illness; right: day 5 of illness)

On day 2 of hospitalization (January 26, 2012), the patient developed dyspnea. Chest auscultation revealed crackles in her right lung. Thoracic ultrasound showed pleural effusion of her right lung, and the chest X‐ray showed bilateral opacities. Acute respiratory distress syndrome (ARDS) was evident based on the rapid progression from unilateral to bilateral pulmonary infiltrate compared to the chest X‐ray image on day 1 (Figure 2). Her oxygen saturation dropped to 80%, requiring intubation and mechanical ventilation.

On day 3 of hospitalization (January 27, 2012), the case‐patient's condition deteriorated and she continued to require mechanical ventilation. Oseltamivir (Tamiflu^®^) 75 mg was enterically administered twice daily via a gastric feeding tube.^8^ Antibiotics and hydrocortisone were given intravenously.

On day 4 of hospitalization (January 28, 2012), her chest examination continued to reveal bilateral crackles. Her oxygen saturation was 40%, demonstrating a rapidly progressive severe hypoxia (SpO2 < 88%, PaO2 < 50 mmHg) since day 2 of hospitalization. The case‐patient was continued on mechanical ventilation and treatment with oseltamivir and antibiotics. Despite intensive supportive care, she died on January 28, 2012.

The tracheal aspirates obtained on the morning of January 27, 2012 tested positive for avian influenza A(H5N1) virus by real time RT‐PCR. After that, virus isolation and genes sequencing were performed, which confirmed the avian influenza A(H5N1) virus of clade 1.1.2.^4^

### 3.2 Clinical course and laboratory data: Newborn baby

**Figure 3:**
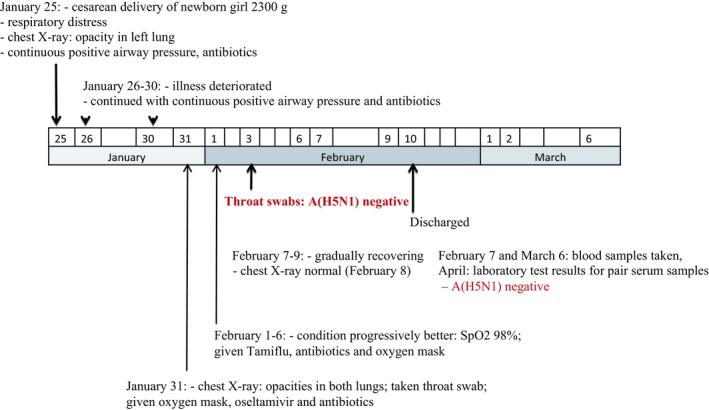
Clinical time line for the newborn of the case‐patient (A[H5N1] positive)

A female newborn baby was delivered by cesarean section on January 25, 2012 of the case‐patient. Indications for cesarean delivery were: breech presentation, oligohydramnios, and fetal distress. The newborn was 48 cm in height, weighing 2300 g (low birth weight) and had a head circumference of 28 cm. The newborn had no apparent congenital defect. The newborn developed respiratory distress secondary to severe intra‐amniotic infection. Her chest X‐ray showed opacity in the left lung field (PO2 36 mmHg, SpO2 65%), and her white blood cell count was 21.6 K/uL. She was initially supported by moist oxygen mask, followed by continuous positive airway pressure (CPAP). Broad spectrum antibiotic eg vancomycin and cefapirin were given; and oseltamivir was administered for 7 days. From day 2 to day 6 of life (January 26 to 30, 2012), the newborn was in a stable but severe condition with continued respiratory distress. On day 7 of life (January 31, 2012) her condition improved, and she was weaned off CPAP to an oxygen mask. From day 8 to day 12 of life (February 1 to 5, 2012), she continued to recover gradually, with normalization of chest X‐ray findings on day 15 (February 8, 2012). She completed a 7‐day course of oseltamivir on February 6, 2012. On day 16 (February 9, 2012) the newborn fully recovered, and had been discharged on day 17 of life (February 10, 2012) (Figure 3).

Throat swab samples were taken from the newborn on January 31, 2012, which were negative for the avian influenza A(H5N1) virus by real time RT‐PCR. Paired serum specimens from the newborn was collected on February 7, 2012 and March 6, 2012 and sent to the influenza laboratory at CDC. The serum specimens were negative for influenza A(H5), A(H3), B, and A(H1) pdm09 viruses.^4^

### 3.3 Epidemiologic investigation

Contact tracing was conducted in the village in the health care facilities where the case‐patient visited. The investigation team listed a total of 73 contacts of which 36 were close contacts and comprised of: seven household members; three neighbors; and 26 health care workers. One close contact had developed mild fever and was isolated in the provincial hospital where throat swab samples were taken and antipyretic was given. A total of 36 throat swab samples were collected from the close contacts (including the hospitalized close contact) on February 1, 2012. It was noted that these close contacts had come into contact with the case‐patient within 10 days after the case‐patient's onset of illness. The results of the throat swab samples for all close contacts revealed negative for the avian influenza A(H5N1) virus.^4^

Local health care workers carried out the monitoring of the contacts and close contacts and monitored adults 14 days after last exposure with the case‐patient and children under the age of 15 years after 21 days of exposure with the case‐patient. Monitoring included daily measurements of temperature. If the temperature was above 38°C or if there were clinical symptoms of acute respiratory disease, then contacts were sent to the hospital for further examination. Oseltamivir was given with dosage of 75 mg (one tablet) per day for 7 days for all close contacts as following the national guidelines.^8^

### 3.4 Case‐patient's exposure history and report of poultry outbreaks

The case‐patient's family raised a flock of 40 backyard chickens, which started dying off on January 8, 2012. Prior to the onset of symptoms, the case‐patient and her mother‐in‐law slaughtered 6 sick chickens between January 17 and 22, 2012. It was noted that the case‐patient had not eaten chicken dishes, had not been exposed to other sick animals, nor handled poultry droppings. Although the mother in‐law had not developed any clinical symptoms, she was monitored and throat swab samples taken. Test results showed that swab samples for the mother in‐law were negative for the avian influenza A(H5N1) virus.

Through interviewing the neighbors and the father of the case‐patient, the investigation team found that between January 8 and 18, 2012, backyard chickens became sick and died off in the hamlet, and these poultry had not received the avian influenza vaccine. Poultry exposure history of the case‐patient was confirmed (Figure 1).

The animal health reports revealed that there had been three avian influenza outbreaks that had occurred in two districts of Sóc Trăng Province and the neighboring Bạc Liêu Province from January 1, 2012 to February 7, 2012. Deaths of the backyard poultry flocks of hamlet, where the case‐patient lived, started from November 28, 2011 until January 25, 2012 (Figure 1).

The animal health authority staff took the throat and rectal swabs from the three poultry flocks nearby the case‐patient's house. Test results showed that these swab samples were positive to the avian influenza A(H5N1) virus by real time RT‐PCR, carried out by the laboratory of the Regional Animal Health Office in Vietnam. These positive swab samples were sent to the National Center for Veterinary Diagnosis in Vietnam for virus culture and sequencing, which confirmed avian influenza A(H5N1) virus of clade 1.1.2.^9^

It was concluded that the avian influenza A(H5N1) virus of clade 1.1.2 that was detected in the poultry samples was highly similar to the virus detected in the case‐patient.^4^, ^9^, ^10^

## 4 DISCUSSIONS

Prior studies have indicated that pregnant women are considered a high‐risk population for influenza A(H1N1) pdm09 and avian influenza viruses and are more likely to develop severe complications and to die, especially when infection occurs in the middle and late trimesters.^6^, ^11^, ^12^, ^13^ The case‐patient, a 36‐week pregnant woman was exposed to sick chickens and was infected with the avian influenza A(H5N1) virus, developed rapid progressive severe pneumonia, lymphopenia, sever hypoxia and increased aminotransferase levels, similar to previously reported avian influenza A(H5N1) patients 6, 14, 15 Common complications of highly pathogenic avian influenza (HPAI) H5N1 virus infection such as primary pneumonia, respiratory failure due to ARDS, and fatal outcome have been seen and recorded in the case‐patient's clinical progress.^16^ The case‐patient died after 6 days from onset of illness, less than the duration period of 9.8 days reported by Shelan et al.^6^

In this case, the case‐patient was exposed to poultry 1 to 5 days prior to onset of illness, whereas the number of days between exposure and onset of illness reported by other studies 14 was 2 to 5 days. Findings from Shelan et al^6^ showed that among 15 A(H5N1) pregnant women the median period from onset of illness to the first consultation was 2 days, and the median number of days from onset of illness to hospital admission was 4 days, whereas for the case‐patient, these figures were 1 day and 3 days respectively. The high awareness of avian influenza and the availability of health care facilities likely encouraged residents of the study community to seek health care more early.

Eight days elapsed from the onset of illness to confirmation of influenza infection, which is the same duration as the median period reported by WHO (8 days).^6^ Findings from the investigation confirmed that sample collection, transport, and laboratory testing systems are working effectively in the Southern region of Vietnam.

The case‐patient received an initial presumptive dose of oseltamivir for 5 days after the onset of illness and before influenza infection was confirmed; however, the WHO technical guidelines recommends that patients with suspected influenza should receive oseltamivir as early as possible to reduce mortality.^17^ Findings by Riberio et al^12^ indicated that antiviral treatment was a protective factor to the A(H5N1) virus when administered within 48 hours of the onset of symptoms.

Recent knowledge has indicated that seasonal influenza vaccination will not prevent infection with avian influenza viruses.^18^, ^19^ However, findings from Henry Dunand et al^20^ showed that the avian influenza H7 reactive antibodies can be found in almost everyone that has the seasonal influenza vaccination. This suggested that the seasonal influenza vaccination may offer some defense against other avian influenza strains not contained in the seasonal vaccine. Therefore, everyone particularly high‐risk groups eg pregnant women, should be vaccinated as a priority.

We described the first reported alive newborn baby delivered by cesarean section from a mother (the case‐patient) infected with the avian influenza A(H5N1) virus during the third trimester of pregnancy, among 14 other pregnant women infected with A(H5N1), but whose fetuses did not survive regardless of the pregnancy trimester.^6^

Previous postmortem studies carried out on pregnant women suggest diffuse systemic dissemination of the A(H5N1) influenza virus including infection in the fetal‐placental membranes,^5^ suggesting that the A(H5N1) virus could be transmitted from mother to fetus across the placenta.^21^ However the newborn baby of this case‐patient was negative of the A(H5N1) virus. The severe clinical symptoms that the newborn developed was neonatal respiratory distress syndrome due to intra‐amniotic infection, which was highly likely caused by her mother's (the case‐patient's) critical condition of viral infection and preterm cesarean delivery at 36 weeks.^22^, ^23^, ^24^

Findings from the in‐depth investigation and laboratory test results from both public health and animal health sectors in Vietnam and the CDC indicated that the source of infection was the case‐patient's backyard poultry and the only recorded source of potential exposure. Highly similar genomic sequence of the same clade of viral strains from the patient and poultry in the case‐patient's neighbor suggested that the source of infection was of clade 1.1.2 avian influenza A(H5N1) virus that was circulating in Southern Vietnam and Cambodia from 2011 to 2014.^9^, ^10^

## 5 CONCLUSIONS

*For early detection of poultry outbreak:* communities should be proactively involved in reporting poultry deaths in a rapid and timely manner. The animal health sectors should strengthen the poultry avian influenza surveillance for early detection and response.

*For diagnosis and case management:* in severe cases, patients should receive early diagnosis of the avian influenza A(H5N1) virus by asking patients about poultry exposures prior to illness. Patients should be hospitalized as soon as possible after diagnosis of suspected avian influenza infection. Oseltamivir should be given as soon as possible (within 48 hours).

*For future research:* intensive studies are needed to better understand the possibility of mother‐to‐child transmission of the avian influenza A(H5N1) virus to protect pregnant women and fetuses. Pregnant women should be prioritized to receive seasonal influenza vaccination given the high risks of severe complication to mothers and their babies.
